# Comparative study of affective temperaments between medical students and humanities students(evaluation by validated temps-a)

**DOI:** 10.1192/j.eurpsy.2021.529

**Published:** 2021-08-13

**Authors:** R. Jomli, J. Ouertani, H. Jemli, U. Ouali, Y. Zgueb, F. Nacef

**Affiliations:** 1 Faculty Of Medicine Of Tunis, university of tunis elmanar, tunis, Tunisia; 2 Mental Health, hospital of jendouba, jendouba, Tunisia; 3 Avicenne, manouba psychiatry, manouba, Tunisia

**Keywords:** student, medicine, TEMPS-A, humanities

## Abstract

**Introduction:**

While the links between cyclothymia and creativity are well documented, the experts have tried to determine whether temperament would influence the major choices of life such as career.

**Objectives:**

The study aims mainly to evaluate the temperaments of a sample of Tunisian students, and to look for the possible correlations between the temperament and the choice of studies.

**Methods:**

The Tunisian version of the TEMPS-A which is a self-evaluation measure to assess affective temperaments was administered to 100 medical students and 100 humanities students.

**Results:**

Student populations differed in their socio-demographic and scholar variables such as age, sex ratio or socio-economic level, choice of studies and their religiosity. The temperamental prevalences were close between our two populations by considering the threshold score Mean +1standard deviation; they ranged between 13 and 18%. Hyperthymic and cyclothymic scores were significantly higher among humanities students (11.38 ± 4.385 versus 9.00 ± 4.192 and 11.96 ± 4.497 versus 9.63 ± 4.499 respectively) and irritable scores were higher in the latter, though not significant (6.45 ± 3.823 versus 5.39 ± 2.998). Depressive and anxious temperament scores were close in both groups. The study showed significant temperament differences within gender, socioeconomic status, high school marks, religiosity and political affiliation.

**Conclusions:**

It is relevant and even necessary to include such studies in the selection of candidates who could adapt to a specific professional field on the basis of objective criteria such as conscientiousness, and privileging profile diversity.

